# Lessons learned from implementation of an electronic decision support tool for hospital-administered pneumococcal vaccinations

**DOI:** 10.1017/ash.2024.380

**Published:** 2024-09-09

**Authors:** Sanchi Malhotra, Rachel Martin-Blais, Ross Pineda, Meganne Kanatani, Ishminder Kaur, Annabelle de St. Maurice

**Affiliations:** 1 Division of Infectious Diseases, Department of Pediatrics, University of California, Los Angeles, CA, USA; 2 Section of Infectious Diseases, Nationwide Children’s Hospital, Columbus, OH, USA; 3 Department of Pediatrics, The Ohio State University, Columbus, OH, USA; 4 Department of Pharmaceutical Services, University of California, Los Angeles, CA, USA

## Abstract

Experts recommend standing orders for hospital-administered vaccines to improve adult immunization rates. We implemented an admission assessment tool to offer pneumococcal vaccine to eligible hospitalized patients. We retrospectively reviewed vaccines for guideline concordance and found that immunization rates increased but less than half of study patients received the correct vaccine.

## Introduction

*Streptococcus pneumoniae* is a leading cause of serious bacterial infections globally, including bacteremia, pneumonia, and meningitis.^1^ Pneumococcal polysaccharide vaccine (PPSV23) was introduced in 1983; however, pneumococcal conjugate vaccines (PCV), first introduced in 2000, had the most significant impact on decreasing disease burden in children and their adult contacts.^1^

In 2014, the Advisory Committee on Immunization Practices (ACIP) recommended PCV13 and PPSV23 for all adults 65 and older for prevention of pneumonia, though this has subsequently been revised.^2^ Data in 2017 showed that although children had a high uptake of PCV (92%), only 69% of adults 65 and older and 24.5% of adults 19–64 years old with high-risk comorbidities had ever received any pneumococcal vaccine (PV).^1^ To improve adult immunization rates, 13 states, including California, legally require acute care facilities to offer PV to adults 65 and older.^3^

Pursuant to these recommendations, our hospital system implemented an electronic decision support (EDS) tool to help nurses (RNs) offer PVs to qualifying inpatients. We aimed to study the accuracy of PVs administered using this EDS tool, hypothesizing that the numerous formulations and changing guidelines for PVs may lead to under-recognized incorrect administration practices.

## Methods

This was a retrospective observational study of patients who received PVs during hospitalization at 2 hospitals within the University of California Los Angeles (UCLA) health system between January 1 and June 30, 2018. The study period was chosen to allow for adequate sampling after consistent implementation of the EDS tool (first initiated in 2013) but prior to changes in the ACIP’s recommendations in 2019. The EDS tool (Supplemental Figure 1) was a nursing clinical application managed by nursing informaticists. It was triggered on admission for patients over age 5 to prompt RNs to conduct a vaccine needs assessment based on age, vaccination history, and comorbidities, with an embedded order if PV was indicated. The default order for the tool was PPSV23; however, it was not obligatory. If PCV13 was indicated, the prescribing provider could order this separately, and the EDS tool would not be used. Whether or not the EDS tool was used was documented for each patient. Duplicate records were excluded.

**Figure 1.**
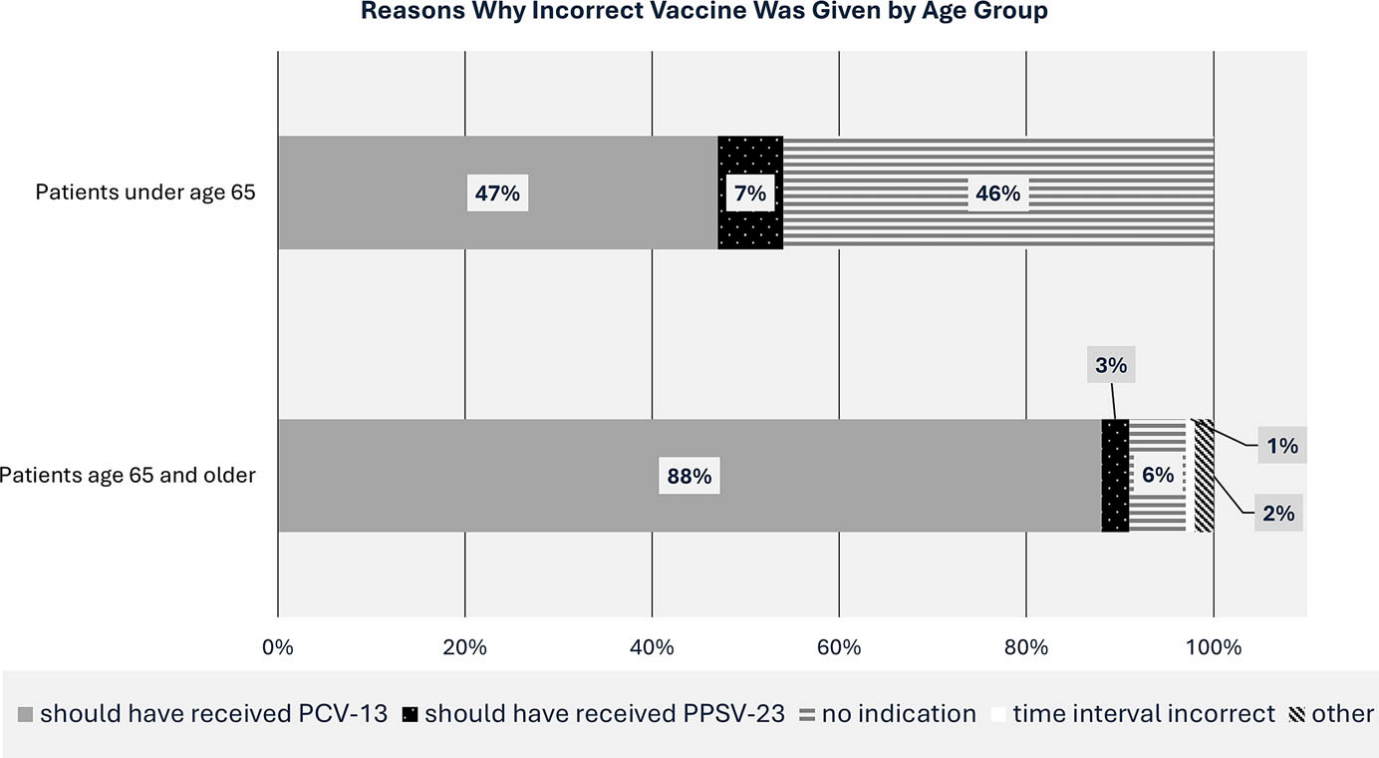
Stacked horizontal bar chart of reasons why an incorrect vaccine was given by age group. Note that 33% of patients under age 65 received an incorrect vaccine and 69% of patients age 65 and older received an incorrect vaccine.

Patient immunization records were examined within the institutional electronic health record (EHR) and any communicating EHRs, including the California Immunization Registry. A chart search for the words “vaccine,” “PCV,” “PPSV,” “pneumococcal,” “Prevnar,” and “Pneumovax” was performed to ensure the inclusion of relevant information not recorded in the dedicated immunizations section.

We reviewed comorbidities to identify qualifying risk factors for pneumococcal disease according to the Centers for Disease Control and Prevention’s (CDC) guidelines.^4^ Determination of vaccine accuracy was based on the 2014 ACIP recommendations in place during the study period.^2^ Each patient’s data and appropriateness determination was reviewed by another author, with a third author resolving any disputes.

This project was exempted by the UCLA Institutional Review Board. All data were recorded in REDcap,^5^ hosted at UCLA. Descriptive statistics, χ^2^ analyses, and odds ratios were calculated to compare categorical variables using IBM® SPSS®.

## Results

We reviewed 461 charts and included 440 unique charts for data analysis. Among this cohort, 72.4% of patients were receiving their first-ever PV. PV administration was initiated by the EDS tool in 79.1% of patients.

The majority of our patients were 65 and older (60.5%, n = 266), and few were under 18 (2.3%, n = 10). The most common comorbidities were chronic heart disease (53%), diabetes mellitus (27.5%), and chronic lung disease (13.2%). Immunocompromised patients comprised 17.5% of the study population.

A correct vaccine was administered 45.2% of the time. Vaccine accuracy varied by age group, comorbidity, vaccine type, and administration via the EDS tool (Table 1). Those without any comorbidities and with chronic heart disease and/or immunocompromised status received the correct vaccine less than half of the time (Table 1).

**Table 1.** Percentage of patients within each demographic/variable that were determined to have received the correct vaccine given their age and prior history

	Correct vaccine was given % (n)	*P*-value
Age group		*P* < 0.01
0–17	80% (8)	
18–39	69.6% (32)	
40–64	64.4% (76)	
>65	31.2% (83)	
Comorbidity^a^		*P* < 0.01
None	22.7% (4)	
Chronic heart disease	40.4% (40)	
Diabetes mellitus	50.6 (42)	
Chronic lung disease^b^	67.7% (42)	
Immunosuppressed^c^	40.3% (31)	
Liver disease/alcoholism	35.3 (36)	
Electronic decision support tool used^d^		*P* < 0.01
Yes	36.8% (128)	
No	77.2% (71)	
Vaccine type		*P* < 0.01
PCV13	84.7% (83)	
PPSV23	33.9% (116)	

a Those with multiple comorbidities were categorized into the “strictest” category (ie, if heart disease and malignancy, characterized as immunosuppressed). There were no patients with cochlear implants or cerebrospinal fluid (CSF) leaks in our study population.

b Including chronic lung disease, asthma, and smoking.

c Including HIV, neutropenia, congenital immunodeficiency, solid organ transplant recipient, congenital or acquired asplenia, malignancy, or iatrogenic immunosuppression.

d The electronic decision support tool was not used if PCV13 was indicated; this would be ordered separately.

Analyzing by an age cutoff of 65 showed a significant difference, with 68.8% of patients 65 and older receiving an incorrect vaccine, compared to only 33.3% under 65 (*P* < .01; OR 4.4; 95% CI, 2.9–6.6). PPSV23 was administered for 77.7% of patients but was the incorrect vaccine in 66.1% of these patients—compared with PCV13, which was incorrect only 15.3% of the time (*P* < .01; OR 10.7; 95% CI, 6–19.6). This is consistent with our data looking at reasons for an incorrect vaccine (Figure 1). “PCV13 should have been given instead” was the most common reason for incorrect administration, especially in the 65 and older cohort. “No vaccine indication” was also a significant reason for those under 65.

The EDS tool successfully vaccinated 79.1% of patients. However, it was administered incorrectly in 63.2% of patients, compared with only 22.8% error seen in those who received a vaccine ordered specifically by the prescribing provider (*P* < .01; OR 5.8; 95% CI, 3.4–9.9). Pharmacists intervened in 35 cases, recommending a correct change 91.4% of the time.

## Discussion

Pneumococcal immunization recommendations are among the most complex of those included in the standard vaccine schedule. As hospital-based vaccination programs become increasingly common, now with new PCV20 and respiratory syncytial virus (RSV) immunizations, in addition to seasonal influenza and severe acute respiratory coronavirus virus 2 (SARS-CoV-2) boosters, as well as the rise of automated systems to standardize healthcare offerings, we felt it was important to share our centers’ prior experience and challenges in vaccine operationalization, particularly surrounding EDS tools.

A significant number of our patients across age ranges were receiving their first-ever PV, underscoring the importance of using any encounter within the healthcare system as an opportune moment to vaccinate. This is consistent with prior studies that showed EDS systems increasing PV rates, but the accuracy of the administered vaccines in these reports was not verified as in our study.^6^ PV decisions are dependent upon interpreting comorbidities and prior vaccinations, which can pose challenges both for individual providers and for EDS systems, particularly when patients may seek care from multiple healthcare settings without universal EHRs. We found that over half of our patients received an incorrect PV, with those 65 and older, those without comorbidities, and those receiving it via the EDS tool to be at particularly high risk.

Identifying and mitigating vaccination errors is of high importance currently. Recent errors were highlighted by the CDC regarding RSV vaccination administration, with children receiving adult vaccines and pregnant people receiving a vaccine type that was not approved in pregnancy.^7^ Errors in the administration of SARS-CoV-2 vaccines also increased after multiple updates to vaccine recommendations in a short period, with errors in age group and schedule being significant factors.^8^ Vaccine administration errors could further undermine the community’s confidence in vaccination.

After reviewing this data, our health system has made steps toward improvement. We are streamlining our EDS algorithm and implementing a pharmacist-led inpatient vaccination protocol. A previous 2016 study showed that implementing a pharmacy-driven PV process improved guideline-concordant care from 42% to 97% of cases.^9^ The health team will also utilize the CDC’s PneumoRecs VaxAdvisor app, created to clarify PV recommendations by age, medical condition, and PV history.^10^

Limitations of this study included the inability to access all outside records, missed comorbidities if problem lists were not up to date, and the inherent limitations of the EDS tool.

## Conclusions

Although vaccinating during hospital encounters is worthwhile, implementing an accurate process is challenging. EDS tools require diligent multidisciplinary reviews and periodic reevaluation of accuracy to be most effective.

## Supporting information

Malhotra et al. supplementary material Malhotra et al. supplementary material

## Supplementary material

For supplementary material accompanying this paper visit http://doi.org/10.1017/ash.2024.380. click here to view supplementary material

## References

[1] Gierke R , Wodi P , Kobyashi M. Pneumococcal Disease. In 2021 *Epidemiology and Prevention of Vaccine-Preventable Diseases* (Pink Book 14th Ed). Centers for Disease Control and Prevention website. https://www.cdc.gov/vaccines/pubs/pinkbook/pneumo.html. Last updated 2021. Accessed Oct 13 2023.

[2] Tomczyk S , Bennett N , Stoecker C et al. Use of 13-valent pneumococcal conjugate vaccine and 23-valent pneumococcal polysaccharide vaccine among adults aged ≥65 years: recommendations of the Advisory Committee on Immunization Practices (ACIP). MMWR 2014;63:822–825. 25233284 PMC5779453

[3] Law section. Accessed October 2, 2023. https://leginfo.legislature.ca.gov/faces/codes_displaySection.xhtml?lawCode=HSC&sectionNum=120392.9.

[4] Centers for Disease Control and Prevention (CDC). Use of 13-valent pneumococcal conjugate vaccine and 23-valent pneumococcal polysaccharide vaccine for adults with immunocompromising conditions: recommendations of the Advisory Committee on Immunization Practices (ACIP). MMWR Morb Mortal Wkly Rep 2012;61:816–819. 23051612

[5] Harris PA , Taylor R , Minor BL , et al. REDCap consortium, the REDCap consortium: building an international community of software partners. J Biomed Inform 2019;95:103208. 31078660 10.1016/j.jbi.2019.103208 PMC7254481

[6] Kapoor S , Sheth HS , DeSilva R , Aggarwal R. Best practice alerts in electronic medical records to improve pneumococcal vaccination in CKD. Am J Kidney Dis 2023;81:245–246. 35987348 10.1053/j.ajkd.2022.06.010

[7] Information on Respiratory Syncytial Virus (RSV) Vaccine Administration Errors in Young Children and Pregnant People. Centers for Disease Control. *January 22*, 2024. https://emergency.cdc.gov/newsletters/coca/2024/012224.html

[8] Hall E , Odafe S , Madden J , Schillie S. Qualitative conceptual content analysis of COVID-19 vaccine administration error inquiries. Vaccines (Basel) 2023;11:254. 36851132 10.3390/vaccines11020254 PMC9961408

[9] Pickren E , Crane B. Impact on CDC guideline compliance after incorporating pharmacy in a pneumococcal vaccination screening process. Hosp Pharm 2016;51:894–900. 28057948 10.1310/hpj5111-894 PMC5199221

[10] PneumoRecs VaxAdvisor: Vaccine Provider App | CDC. Published September 26, 2023. Accessed October 2, 2023. https://www.cdc.gov/vaccines/vpd/pneumo/hcp/pneumoapp.html

