# Supplementary figures and images for: Lessons learned from implementation of an electronic decision support tool for hospital-administered pneumococcal vaccinations
Source: Antimicrob Steward Healthc Epidemiol. 2024 Sep 9;4(1):e117. doi: 10.1017/ash.2024.380 (PMC11384155; doi:10.1017/ash.2024.380)

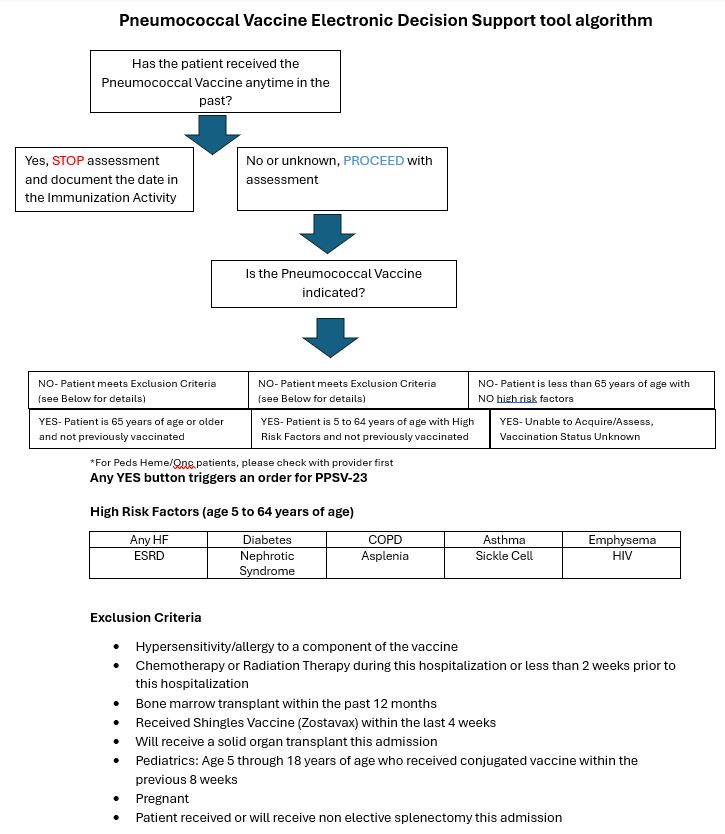

Supplement: Malhotra et al. supplementary material [file S2732494X24003802sup001.zip › SuppFigure1_ASHE.JPG]
